# *Brachyspira pilosicoli *bloodstream infections: Case report and review of the literature

**DOI:** 10.1186/1476-0711-7-19

**Published:** 2008-09-25

**Authors:** Lilia Bait-Merabet, Arnaud Thille, Patrick Legrand, Christian Brun-Buisson, Vincent Cattoir

**Affiliations:** 1 Service de Bactériologie-Virologie-Hygiène, C.H.U. Henri Mondor, Assistance Publique-Hôpitaux de Paris, Créteil, France; 2 Service de Réanimation Médicale, C.H.U. Henri Mondor, Assistance Publique-Hôpitaux de Paris, Créteil, France

## Abstract

*Brachyspira pilosicoli *is the etiologic agent of human and animal intestinal spirochetosis and is rarely implicated as a cause of bacteremia. Here, we describe the case of a *B. pilosicoli *spirochetemia in a 53-year-old male patient suffering from cardiogenic shock. This fastidious bacterium was isolated from blood, likely after translocation from the intestinal tract. Blood cultures were positive after 5 days of incubation (one day after the patient's death), highlighting the problem of the recovery of such type of fastidious bacterium. Identification was achieved by molecular methods (16S rRNA sequencing). A review of the English literature found only 8 cases of bacteremia caused by *B. pilosicoli*, mostly in immunocompromised or critically ill patients. Finally, difficulties in rapid and accurate diagnosis of *B. pilosicoli *bloodstream infections, in vitro antimicrobial susceptibility of human clinical isolates, and therapeutic options are discussed.

## Introduction

The genus *Brachyspira*, first described in 1982, belongs to the order of the Spirochaetales (like *Treponema*) and includes seven species to date: *B. aalborgi*, *B. alvinipulli*, *B. hyodysenteriae*, *B. innocens*, *B. intermedia*, *B. murdochii*, and *B. pilosicoli *. Morphologically, they are coiled Gram-negative bacilli (2 to 11 μm long; 0.2 to 0.5 μm in diameter) and are motile due to the presence of flagella (8 to 30). These anaerobic intestinal spirochetes colonize the large intestine of humans and many animal species, and several species may cause diarrhea and other gastrointestinal diseases in these hosts [1]. Although *Brachyspira *(formerly *Serpulina*) *pilosicoli *is the etiologic agent of porcine intestinal spirochetosis [2], *B. pilosicoli *and *B. alborgi *are both described in humans and are considered as agents of human intestinal spirochetosis (HIS) [3,4]. In addition, *B. pilosicoli *was also isolated from patients with bacteremia, termed spirochetemia [5-7]. In this report, we describe a case of spirochetemia due to *B. pilosicoli *in a peculiar clinical context (cardiogenic shock) and review the literature.

## Case report

A 53-year-old man, obese and smoker, was admitted to the cardiology unit of our hospital for shock associated with a third-degree atrioventricular block. Upon admission, five sets of peripheral blood cultures were systematically drawn; however, antibiotics were not initiated because the patient was afebrile. In the cardiology unit, he had two episodes of ventricular fibrillation treated by external cardioversion. Coronarography was performed and showed an occlusion of the right main coronary artery that disappeared spontaneously whereas the left main coronary artery was normal. Ventriculography was normal and aortography revealed the absence of aortic dissection. Due to persisting cardiogenic shock, the patient was then transferred to the medical intensive care unit on the same day where he received inotropic drugs. Because of leukocytosis (white blood cell count of 16,000 cells/mm^3^) and hypothermia (body temperature at 35.3°C), an antibiotic treatment with cefotaxime (3 g daily) was started. A transoesophageal echocardiography revealed right atrial and ventricular massive dilatation and an acute right ventricular dysfunction. A diagnosis of cardiogenic shock due to transient occlusion of the right main coronary artery was made. In the unit, his evolution was rapidly unfavourable with the occurrence of multiple and repeated episodes of ventricular fibrillation. The patient died four days later in a context of multiple organ failure associated with persistent cardiogenic shock and right ventricular failure.

The day after of the patient's death (i.e. five days of incubation), four out of five BacT/Alert^® ^blood culture anaerobic bottles (bioMérieux, Marcy l'Etoile, France), yielded a Gram-negative spiral-shaped bacterium, suggesting a spirochete. After three days, the isolate was subcultured on 5% horse blood-containing agar plates under anaerobic conditions at 37°C and the growth appeared as a thin film with weak β-hemolysis. Phenotypically, all biochemical tests except hippurate were negative using the API 20 ANA and ID 32 ANA strips (bioMérieux). In addition, the isolate yielded a positive reaction for oxidase test and negative reactions for catalase and indole production tests.

Accurate identification of the clinical isolate was achieved by 16S rRNA sequence analysis, as previously described [8]. The 16S rRNA nucleotide sequence of the clinical isolate was then compared to deposited sequences available from GenBank using the BLAST program. The sequence of the clinical isolate was 100% identical with those of *B. pilosicoli *ATCC 51139 and P43/6/78 strains (GenBank accession no. AY155458 and U14927, respectively).

Disk diffusion susceptibility testing was performed on Mueller-Hinton agar supplemented with 5% defibrinated horse blood according to the recommendations of the Antibiogram Committee of the French Society for Microbiology . The strain was fully resistant to penicillin, amoxicillin, ticarcillin and piperacillin by producing a β-lactamase (cefinase test positive). It was also resistant to erythromycin, spiramycin, rifampin, vancomycin and colistin, and susceptible to amoxicillin/clavulanate, piperacillin/tazobactam, cefoxitin, cefotaxime, imipenem, clindamycin, pristinamycin, tetracyclin, chloramphenicol and metronidazole.

## Discussion

In this report, we describe a case of *B. pilosicoli *bloodstream infection in a context of cardiogenic shock. To the best of our knowledge, only a few cases of bacteremia caused by this species have been reported in the literature.

Both *B. aalborgi *and *B. pilosicoli *are implicated as causal agents of HIS, an infestation by spirochetal microorganisms on the surface of the large intestinal mucosa first described in 1967 [9]. The two species are zoonotic since they have been isolated from the feces of non-human primates or other animals, such as dogs and birds [1]. Little is known about the transmission of these spirochetes to humans but it seems likely to occur by fecal-oral route, e.g. after exposure to colonized/infected human or animals, their feces, or contaminated water [1]. The incidence of *B. aalborgii *and *B. pilosicoli *infections in humans is poorly investigated, but the use of species-specific PCR assays should allow the prompt and accurate detection of the spirochetes in clinical samples [4]. Prevalence of IS varied considerably across geography and immune conditions. Indeed, the prevalence of *B. pilosicoli *in rectal biopsies is reported to be 2–7% in Western countries, 11–34% in less developed countries, and up to 54% in homosexual men and HIV-infected patients [3,4,10,11]. It is noteworthy that intestinal colonization with *B. pilosicoli *appears high (prevalence > 30%) in specific populations, such as Australian Aboriginal children, Gulf Arabs, and villagers in Papua New Guinea [4,12,13]. In certain conditions, particularly immunosuppression, *B. pilosicoli *may translocate from the large intestine to blood (causing spirochetemia) since the adherence of the spirochete to the colonic epithelium is likely increased in these situations. Nevertheless, only 8 cases of *B. pilosicoli *bacteremia have been previously described, including 6 cases from France in patients older than 50-years and having a severe underlying disease that led to death in four cases [5] (Table 1). Two other cases have been reported, the first from the United States in a Californian HIV-infected patient with a Kaposi's sarcoma [6], and the second from Greece in an immunocompromised patient suffering from a non-Hodgkin's lymphoma [7] (Table 1). Out of these 9 cases of bacteremia (including the present case), 8/9 (89%) were male and 9/9 (100%) were older than 50 with a severe underlying disease and/or an immunosuppression leading to death in 66.7% of cases (6/9). In our patient, *B. pilosocoli *blood isolate is likely to have originated in the ischemic intestinal tract during cardiogenic shock since, in such cases, blood isolates are closely related to fecal isolates [14]. Translocation of bacteria or endotoxin from the intestinal tract commonly occurs in debilited patients as a result of overgrowth of Gram-negative bacteria, impaired host defenses, or injury to the gut mucosa resulting in increased mucosal permeability [6].

**Table 1:** Characteristics of 9 confirmed cases of bacteremia caused by *B. pilosicoli*

**Ref**.	**No. of cases**	**Age**	**Sex**	**Underlying disease**	**Treatment/Outcome**
[5]	6^a^	77 69	F M	Stroke/hemiplegia Stroke/hemiplegia	Death Death
		55	M	Alcoholic/acute intoxication by ethylene glycol	Death
		69	M	Acute peritonitis (necrosis of caecum)	Operation/recovery
		61	M	Severe arteriopathy	Vascular operation/death
		52	M	Myeloma/alcoholic	Antibiotic therapy/chemotherapy
[6]	1	-^b^	M	AIDS/Kaposi's sarcoma	-^a^
[7]	1	78	M	Non-Hodgkin's lymphoma/alcoholic/tabagism	Antibiotic therapy/death
This report	1	53	M	Tabagism, cardiogenic shock	Death

^a ^Previously classified as *B. hyodysenteriae*, organisms were accurately identified as strains of *B. pilosicoli *in reference 6; ^b ^unknown

Although spirochetemia remains very rare, incidence of *B. pilosicoli *bacteremia is probably more common than clinically appreciated since detection time in automated growth detection systems often exceed 5 days under anaerobic conditions [15]. In addition, subculture of *B. pilosicoli *from blood culture media is also fastidious (generally from 3 to 5 days at 37°C) and phenotypic identification to the species-level cannot be achieved by using available commercial kits [2]. By contrast, the use of genotypic identification by sequencing the 16S rRNA gene allows clear identification to the species-level. Therefore, the use of PCR assays using specific primers for the 16S rRNA gene to directly identify the spirochetes recovered in blood should be strongly recommended.

Even if the diagnosis is often delayed like here, preferred treatment of *B. pilosicoli *infections remains metronidazole. Indeed, all isolates of *B. pilosicoli *recovered from humans (n = 123) were susceptible to metronidazole [16]. Although β-lactam antibiotics are spirocheticidal, our *B. pilosicoli *strain was resistant to penicillins due to production of a β-lactamase. A novel class D β-lactamase (OXA-63), recently identified in the chromosome of *B. pilosicoli *BM4442, may explain this phenotype [17]. Unfortunately, prevalence of *bla*_OXA-63 _gene has not been studied; however, an *in vitro *antimicrobial susceptibility survey on 139 *B. pilosocoli *clinical isolates showed a 54.7% susceptibility rate to amoxicillin, suggesting that resistance to penicillins is common [16]. As previously reported, our strain was also resistant to erythromycin, but susceptible to third-generation cephalosporins, carbapenems, tetracyclin and chloramphenicol, [16]. The resistance to macrolides in *B. pilosicoli *is due to point mutations in positions 2058 and 2059 of the 23S rRNA gene [18].

## Conclusion

Our report confirms that *B. pilosicoli *may cause bacteremia in immunocompromised and/or critically ill patients. The contribution of the spirochetemia to the lethal outcome of our patient remains unclear, since he was debilitated with an underlying poorly controlled cardiogenic shock, and received effective therapy. However, rapid and accurate diagnosis methods (e.g. PCR assays) should be employed to identify the etiologic microorganism and help adapt antibiotic therapy for such infections.

## Competing interests

The authors declare that they have no competing interests.

## Authors' contributions

Each author acknowledges that he has contributed in a substantial way to the work described in the manuscript and its preparation. All authors read and approved the final manuscript.

## Consent

We did informed consent for the patient about publishing the manuscript of Annals of Clinical Microbiology and Antimicrobials. Written informed consent was obtained from the patient for publication of this case report and any accompanying images. A copy of the written consent is available for review by the Editor-in-Chief of this journal.
